# Correction: Pressure- and temperature-driven local structural stability and 5f/d hybridization in a metastable U–5.28 wt% Nb alloy

**DOI:** 10.1039/d6ra90125e

**Published:** 2026-09-21

**Authors:** Rongqiang Yao, Rui Zhou, Xudong Tan, Yilin Fang, Zexin Jiang, Jintao Wang, Yuanyuan Wang

**Affiliations:** a Rocket Force University of Engineering Xi'an 710025 China wangjintaolove@126.com; b School of Materials Science and Engineering, Key Laboratory of Materials Modification by Laser, Ion and Electron Beams (Ministry of Education), Dalian University of Technology Dalian 116024 China yuanyuanwang@dlut.edu.cn

## Abstract

Correction for ‘Pressure- and temperature-driven local structural stability and 5f/d hybridization in a metastable U–5.28 wt% Nb alloy’ by Rongqiang Yao *et al.*, *RSC Adv.*, 2026, https://doi.org/10.1039/d6ra06126e.

The authors regret that incorrect references were identified in this paper following self-inspection of the reference list post-publication. Bibliographic metadata errors were found for ref. 29–31. The correct references are given below as ref. 1–3. The in-text superscript citation markers remain unmodified. This correction does not change any results or conclusions of the original article.

The Royal Society of Chemistry apologises for these errors and any consequent inconvenience to authors and readers.
